# Low-cost HPV testing and the prevalence of cervical infection in asymptomatic populations in Guatemala

**DOI:** 10.1186/s12885-018-4438-y

**Published:** 2018-05-15

**Authors:** Hong Lou, Eduardo Gharzouzi, Sarita Polo Guerra, Joël Fokom Domgue, Julie Sawitzke, Guillermo Villagran, Lisa Garland, Joseph F. Boland, Sarah Wagner, Héctor Rosas, Jami Troxler, Heidi McMillen, Bailey Kessing, Enrique Alvirez, Miriam Castillo, Hesler Morales, Victor Argueta, Andert Rosingh, Femke J. H. B. van Aerde-van Nunen, Griselda Lopez, Herbert M. Pinedo, Mark Schiffman, Michael Dean, Roberto Orozco

**Affiliations:** 1 Laboratory of Translational Genomics, Division of Cancer Epidemiology and Genetics, Leidos Biomedical Research, Inc., National Laboratory for Cancer Research, Gaithersburg, MD USA; 2 Instituto de Cancerologia, 6ª Avenida 6-58, Zona11, Guatemala City, Guatemala; 3 Cancer Genetics Branch, Gaithersburg, MD USA; 4 Laboratory of Translational Genomics, Division of Cancer Epidemiology and Genetics, National Cancer Institute, Gaithersburg, MD USA; 5 Department of Gynecology and Obstetrics, Hospital General San Juan de Dios, Guatemala City, Guatemala; 6 Cancer Research Technology Program, Leidos Biomedical Research, Inc., National Cancer Institute, 8560 Progress Drive, Frederick, MD 21701 USA; 7 Hospital Central Universitario “Dr. Antonio M Pineda”, Lara State, Barquisimeto, Venezuela; 8 Department of Pathology, Hospital General San Juan de Dios, Guatemala City, Guatemala; 9 Medical Laboratory Services, Willemstad, Curaçao; 10 Fundashon Prevenshon, Willemstad, Curaçao

**Keywords:** HPV, Cervical cancer, Guatemala, Prevalence, Screening, Real-time PCR

## Abstract

**Background:**

A low cost and accurate method for detecting high-risk (HR) human papillomavirus (HPV) is important to permit HPV testing for cervical cancer prevention. We used a commercially available HPV method (H13, Hybribio) which was documented to function accurately in a reduced volume of cervical specimen to determine the most prevalent HPV types and the distribution of HPV infections in over 1795 cancer-free women in Guatemala undergoing primary screening for cervical cancer by cytology.

**Methods:**

HR-HPV detection was attempted in cervical samples from 1795 cancer-free women receiving Pap smears using the Hybribio™ real-time PCR assay of 13 HR types. The test includes a globin gene internal control. HPV positive samples were sequenced to determine viral type. Age-specific prevalence of HPV was also assessed in the study population.

**Results:**

A total of 13% (226/1717) of women tested HPV+, with 78 samples (4.3%) failing to amplify the internal control. The highest prevalence was found in younger women (< 30 years, 22%) and older ones (≥60 years, 15%). The six most common HR-HPV types among the 148 HPV+ typed were HPV16 (22%), HPV18 (11%), HPV39 (11%), HPV58 (10%), HPV52 (8%), and HPV45 (8%).

**Conclusions:**

In this sample of cancer free women in Guatemala, HPV16 was the most prevalent HR type in Guatemala and the age-specific prevalence curve peaked in younger ages. Women in the 30-59-year age groups had a prevalence of HR-HPV of 8%, however, larger studies to better describe the epidemiology of HPV in Guatemala are needed.

**Electronic supplementary material:**

The online version of this article (10.1186/s12885-018-4438-y) contains supplementary material, which is available to authorized users.

## Background

Across the world, women living in poverty suffer disproportionally from cervical cancer (CC). In Guatemala, CC is a leading cause of cancer in women (1530 cases/year, Age-standardized rate (ASR) 31/100,000) resulting in an estimated 717 deaths) (Globocan 2012). In the Instituto de Cancerología (INCAN), the main adult oncology hospital in Guatemala, over 40% of women diagnosed with malignancies have advanced CC, requiring costly management that often has a poor outcome [1]. Therefore, a focus on prevention is important.

There is overwhelming evidence that persistent infection with specific types of HPV is the main cause of CC [2, 3]. HPV types that are classified as established carcinogens are HPV16, 18, 31, 33, 35, 39, 45, 51, 52, 56, 58, 59, and possibly 68 [4]. While prophylactic vaccination of adolescents and possibly young adults is the ultimate preventive strategy, screening will remain important for decades to come. Cytology-based screening has been associated with a major reduction in the incidence and mortality of the disease in developed countries [5]. However, cytology is either unavailable or poorly conducted in most low-income countries [6].

In most populations, HPV incidence peaks in women in their late teens or early 20s [7–9] following the average age of first sexual intercourse. Incidence and prevalence typically decline from age 30-45 [10]. A second peak is often observed in postmenopausal women, possibly related to immune senescence and escape from long-term cell-mediated immune control of infections acquired earlier in life [11, 12]. HPV DNA testing has been proposed as an alternative to cytology in women older than 25-30 years, when prevalence declines and predictive value (for signaling risk of precancer) of a positive HPV test increases. [13–15]. However, commercially available tests are typically expensive and require sophisticated equipment [16, 17]. The use of HPV assays targeting lower-resource settings would be useful for CC prevention in such settings, which contribute most of the world CC burden.

This study sought to assess the performance of a validated low-cost HPV assay (H13, Hybribio) in the setting of a low and middle income country [18]. We determined the prevalence of HR-HPV infection in women attending screening clinics in Guatemala.

## Methods

### Study populations

To explore the epidemiology of HPV in Guatemala using H13 (Hybribio Ltd., Hong Kong), a total of 1795 samples were obtained from asymptomatic sexually active women undergoing routine primary screening with Pap smear at hospital-based screening clinics in Guatemala, after obtaining informed written consent. The participants were recruited from women attending cervical cytology screening programs at public hospitals in two urban areas (Guatemala City, and the coastal city of Puerto Barrios), and were tested only one time. Most women live near the respective hospital and so this is a largely urban sample. A questionnaire on reproductive and socio-demographic characteristics was administered by trained personnel on the Guatemalan subjects [19]. The samples were collected by a medical practitioner using a Dacron swab placed in a tube containing 3 ml of Scope mouthwash, maintained at 4° C and transported at room temperature, and stored at -20oC [20].

### HPV testing

The detection of HPV was performed using a Hybribio Assay that detects the 13 high-risk HPV types by real-time PCR. The performance of the assay was previously evaluated in a clinical validation study [18]. A cell lysate was prepared per the manufacturer’s instructions and real-time PCR performed with 1 ul of lysate, 8.75ul of Master mix (containing probes and primers) and 0.25ul of Taq DNA polymerase. Reactions were incubated for 2 min at 50o, 10 min at 95o and 45 cycles of 10 s, 95o; 50 s 58o with data collection at each cycle during the 58o phase on an ABI 9700 or 7000. Each 96-well plate included four HPV+ controls: CC cell line DNA from HeLa (HPV18), CaSki (HPV16), MS751 (HPV45) DNA and positive control DNA from the Hybribio Assay kit; HPV- controls included C33A DNA and water. The kit contains an internal control (human globin gene), and samples that failed to amplify this internal control (78 samples, 4.3%) were excluded from further analysis.

**Fig. 1:**
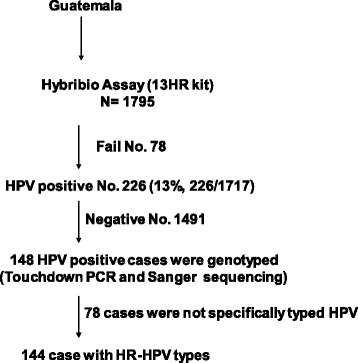
Age specific prevalence of HR-HPV by age group in Guatemala. The prevalence of HR-HPV in asymptomatic women in Guatemala is displayed by age group. Error bars show 95% confidence intervals for HPV+ percentage. The number of total women in each age group is shown in Table 2

To determine HPV type by sequencing, HPV positive samples were subjected to Touchdown PCR and DNA sequencing (Fig. 1). Samples in which the internal control (IC) did not amplify on two separate attempts were excluded (171 samples). The sensitivity of the Touchdown PCR was determined by a series of 10-fold dilutions of DNA from HPV+ and HPV- cell lines using Broad-Spectrum (BS) GP5+ and GP6+ Primers (BSGP5+/6+) (150 bp) (Additional file 1: Figure S1) [21]. The HPV+ samples were amplified by using 400 nM BSGP5+/6+. Briefly, 10 min denaturation step at 95 °C was followed by 40 cycles of amplification. Each cycle included denaturation at 94 °C for 20 s, annealing at 38 °C for 30 s, and elongation at 71 °C for 80 s. The final elongation step was 5 min. The ramping rates were adjusted as described [22]; 1.8 °C/cycle from 74 °C to 38 °C in first 20 cycles. Each experiment included HPV+ and HPV- controls and a sample lacking template DNA (Additional file 1: Figure S2). The PCR products were subjected to Sanger Sequencing on an ABI3730XL. Sequences were analyzed by assembly and trimming in SeqMan (DNASTAR, Madison, WI) followed by BLAST search (NCBI). Samples with inconclusive Sanger sequence were repeated with a next-generation sequencing method (S. Wagner, and J. Boland, manuscript in preparation).

### Statistical analyses

Statistical analyses on the samples were performed to determine age-specific HPV prevalence, comparing the age groups with the Pearson Chi-square test using GraphPad Prism version 7 for Windows. *P* < 0.05 was regarded as statistically significant. We performed analyses of association between HPV infection and age.

## Results

To evaluate the minimum required assay volume of the H13 test we tested the standard 20 ul and 10ul and determined that a 10ul real-time PCR volume gave equivalent results (Table 1); whereas 5ul did not (data not shown). In a separate study, we determined that the H13 test give high concordance with the Qiagen HC2 and Onclarity assays, and has good clinical accuracy compared to histologic diagnosis [18].

**Table 1:** Comparison of the H13 Assay 10 μl and 20 μl volumes

		Input DNA (5 ng/μl)		13HR HPV Types (FAM)	Internal Control (JOE)	HPV Status
Sample	Total Volume (μl)	Volume (μl)	Input DNA (ng)	Ct	Ct	
HPV positive controls						
CaSki (HPV16)	20	2	10	15.74	27.26	+
	10	1	5	16.15	28.61	+
HeLa (HPV18)	20	2	10	22.26	26.23	+
	10	1	5	23.03	26.26	+
MS751 (HPV45)	20	2	10	26.90	29.79	+
	10	1	5	27.96	29.91	+
ME180 (HPV39/18)	20	2	10	27.61	25.99	+
	10	1	5	27.72	25.96	+
Unknown samples from cell lysate						
S1 DD015959	20	2		ND^a^	24.05	–
	10	1		ND	24.80	–
S2 DD015960	20	2		17.60	27.59	+
	10	1		18.02	28.10	+
S3 DD015963	20	2		16.85	20.78	+
	10	1		17.20	21.30	+
S6 DD015964	20	2		16.01	22.05	+
	10	1		16.80	22.70	+
HPV negative control (C33A)	20	2	10	ND	25.66	–
	10	1	5	ND	25.04	–
Controls from kit						
Positive control (Kit)	20	2		23.88	23.39	+
	10	1		25.07	25.68	+
Negative control (Kit)^a^	20	2		ND	ND	–
	10	1		ND	ND	–

DNA samples from positive and negative controls along with four unknown samples were assayed using the H13 kit using both 10 and 20ul volumes. The Ct values are shown as well as the interpretation of HPV status

*ND* not detected

^a^the negative control is DNase-free distilled water from the kit

**Fig. 2:**
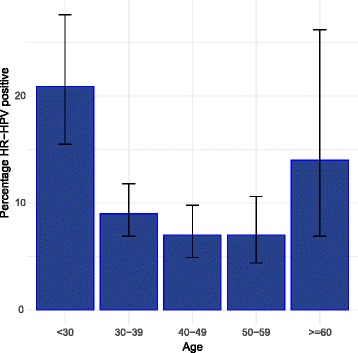
Flow chart of participants and associated HPV test outcomes. The flow chart of samples through the analyses is shown

To apply the H13 test to samples from a low-middle income country, we used the test to determine the prevalence and distribution of HPV types in Guatemala. We recruited asymptomatic women from the general population at hospital clinics performing primary screening for cervical cancer by cytology. The women with available data on age ranged in age from 17 to 79 years attending clinics in Guatemala City and the city of Puerto Barrios. To determine the prevalence of HPV infection, 1717 subjects were tested (Fig. 1). The overall prevalence of HPV infection was 13% (226/1717) (Table 3). To understand the age specific prevalence of HPV infection, the women were divided into 6 age groups (Table 3). HPV prevalence ranged from 21% in the < 30 group to 7.0% to 9.0% in the 30-59 age groups and 14% in the ≥60 group. Age specific prevalence for HPV was significantly higher in the younger age group (< 30) than the 30-59-year age groups (Table 2 and Fig. 2).

**Table 2:** Analysis of age as related to 13HR HPV detection by the H13 assay

Age group	HPV-No.	HPV+ No.	HPV+ (%) (95% CI)	OR	95% CI of OR	*P* value
< 30	136	36	21 (16-28)	Ref		
30-39	474	47	9 (7-12)	0.43	0.27 to 0.69	0.0004
40-49	401	30	7 (5- 10)	0.33	0.20 to 0.56	< 0.0001
50-59	244	18	7 (4-11)	0.33	0.18 to 0.60	0.0003
≥60	43	7	14 (7-26)	ns		

Subjects with available data on age (N-1298) were used to determine the effect of age group on HR-HPV infection using the under 30 age group as a reference. The 95% CI of HPV+ (%) was calculated with the Binomial (Clopper-Person) exact method based on the beta distribution

*Ref* reference, *ns* not significant

HPV typing was successful in 148 out of 226 HPV+ samples and the 13 HR-HPV types 16, 18, 31, 33, 35, 39, 45, 51, 52, 56, 58, 59, 68 were detected in 143 subjects (Table 3.) (143/226). Of the 13 HR types, HPV16 (22%, 32/148), HPV18 (11%, 16/148) accounted for 32% (48/148) and HPV39, 58, 45, 52 combined accounted for 37%, 55/148). Types considered to convey very low risk (Unk), HPV67 and 74 were detected in 1% (Table 3).

**Table 3:** Prevalence of HR-HPV types in Guatemalan women

	HPV type	No.	Percentage in 13HR (%)
13 HR types	16	32	22%
	18	16	11%
	31	4	3%
	33	2	1%
	35	6	4%
	39	16	11%
	45	12	8%
	51	5	3%
	52	12	8%
	56	6	4%
	58	15	10%
	59	10	7%
	68	7	5%
Total		143	

HPV type was determined from Sanger sequence after touch-down PCR or next-generation sequencing. Also detected were individual samples with HPV30, HPV42, HPV52, HPV55, HPV66, HPV67, HPV70, HPV73, HPV86, HPV114 and two samples each with HPV53, HPV71 and HPV74. Multiple infections could only be assessed in 27 samples positive by the next-generation sequencing assay; 6/27 samples (22%) had multiple infections but only 1/27 (4%) had multiple HR infections

## Discussion

Few studies of HPV prevalence have been described in Guatemala, even though this country has one of the highest incidences of HPV related diseases and mortality in Latin America [1, 23, 24]. However, our study has the advantage of collecting samples from a screening population using a low-cost HPV screening method. The overall prevalence of HR HPV infection in this study was 13% for Guatemala, similar to rural Costa Rica [25] and other countries (25–29%) [7, 26, 27].

HPV16 is the most common type found in women with cervical cancer worldwide, and is among the most common high-risk type in cancer-free women [26, 28–32], and the same is true in Guatemala. The combined prevalence of HPV16, and HPV18 was highest in the youngest age groups in this country. A similar prevalence of HPV16 and 18 was reported in other studies [33] as well as in Guatemalans with CC [19]. In addition to HPV16 and 18, 15 other HPV types were observed frequently in our study, most notably HPV39, 58, 45, and 52. The relatively small number of women < 30 years of age had the highest prevalence, while the HPV prevalence decreased markedly with increasing age, up to age 60. This trend has been observed in Costa Rica and other countries [7–9, 30].

Numerous studies support HPV testing as the most sensitive primary screening method for CC. However, there are few HPV tests that are affordable for LMICs. The Hybribio H13 test is a sensitive test that costs $6/assay at the recommended 20ul volume. Commercial tests in Guatemala cost between $100-210, out of the range of practical use. Mexico carried out a large screening program with the hybrid capture assay (HC2, Qiagen, Germantown, MD) at an approximate cost of $11 per test, but many poor and rural areas remain unscreened. A method has been developed with support from donor and non-profit foundations, called careHPV (Qiagen). This system has been rigorously tested in China, India and in pilot programs in other areas [34] and is under further evaluation in several Latin American countries. It is unclear whether it can be scaled up to cover an entire whole population. Thus, there is still considerable discussion on the most effective strategies for managing HPV+ women in different economic and cultural settings [35–37].

We sought to establish a method that would be cost effective, and use only equipment available in many molecular biology laboratories. We employed a validated storage buffer, mouthwash containing 15% ethanol, which costs $0.01 per sample with fewer shipping requirements than methanol based buffers [20]. The Hybribio Assay under the conditions we employed (10ul reaction volume), costs about $3/assay (Table 1). While Hybribio requires a real-time PCR instrument, we have purchased used ABI7000 instruments for under $1000, and found that some Latin American hospitals and clinical laboratories have real-time PCR instrumentation.

Our study has several limitations that might affect our conclusions. We used a Hybribio assay that detects only 13 HR types. In addition, we attempted to sequence all positive samples to determine type. In a portion of samples (4.3%), we had a failure to detect the IC indicating a failure in sample collection, preservation or storage. Most of these samples were negative using both Hybribio and a touchdown PCR method (data not shown). This could indicate a limitation to using Scope mouthwash as a preservative. We have limited histopathology or other outcome data on the women and have not demonstrated that Hybribio Assay is effective in CC prevention in Guatemala. However, the manufacturer reports data on a comparison with Qiagen (with > 95% agreement with an FDA approved test), the test passed two WHO proficiency trials and has been used in a study of 48,559 women in China [11].

## Conclusions

In conclusion, we have used the Hybribio H13 test, an affordable alternative for HPV screening to determine that the high-risk HPV prevalence is 13% in a population of asymptomatic women in Guatemala. The distribution of HPV types is typical of other countries and the highest HPV prevalence is in youngest age groups (< 30). This low-cost approach to detect HPV could be employed in other countries planning to introduce HPV screening to reduce the burden of CC.

## Additional files

Additional file 1: **Figure S1.** Amplification of HPV positive and negative cell line genomic DNA by Touchdown PCR using BS GP5+/6+ primers. A dilution series of an HPV positive cell line (HeLa), HPV negative (HEK293) and HPV-status unknown cell lines were amplified by touchdown PCR with both HPV primer sets and a globin primer pair, and run on 1.5% agarose gels. **Figure S2.** Touchdown PCR from swab cell lysate, (A): Lane 1.- Lane 4 HPV positive control with input DNA 1 ng, 0.1 ng, 0.01 ng and 0.001 ng; Lane 5. HPV negative control (cell line); Lane 6. no template control. Lane 7 to Lane 16 indicate unknown swab samples. M indicate the DNA ladder marker. Touchdown PCR from HPV positive swabs by Hybribio Assay (B): Lane 1. – Lane 18 are swab cell lysate; Lane 19. HPV positive control (cell line); Lane 20. HPV negative control (cell line); Lane 21. no template control. The samples were amplified with the BSGP5+/6+ primers. (DOCX 2548 kb)

## Abbreviations

CC: Cervical cancer
HPV: Human papilloma virus
HR: High-risk
INCAN: Instituto de Cancerología
PCR: Polymerase chain reaction
