# Correction to: Low-cost HPV testing and the prevalence of cervical infection in asymptomatic populations in Guatemala

**DOI:** 10.1186/s12885-020-6672-3

**Published:** 2020-02-28

**Authors:** Hong Lou, Eduardo Gharzouzi, Sarita Polo Guerra, Joël Fokom Domgue, Julie Sawitzke, Guillermo Villagran, Lisa Garland, Joseph F. Boland, Sarah Wagner, Héctor Rosas, Jami Troxler, Heidi McMillen, Bailey Kessing, Enrique Alvirez, Miriam Castillo, Hesler Morales, Victor Argueta, Andert Rosingh, Femke J. H. B. van Aerde-van Nunen, Griselda Lopez, Herbert M. Pinedo, Mark Schiffman, Michael Dean, Roberto Orozco

**Affiliations:** 1 Laboratory of Translational Genomics, Division of Cancer Epidemiology and Genetics, Leidos Biomedical Research, Inc., National Laboratory for Cancer Research, Gaithersburg, MD USA; 2 Instituto de Cancerologia, 6ª Avenida 6-58, Zona11, Guatemala City, Guatemala; 3 Cancer Genetics Branch, Gaithersburg, MD USA; 4 Laboratory of Translational Genomics, Division of Cancer Epidemiology and Genetics, National Cancer Institute, Gaithersburg, MD USA; 5 Department of Gynecology and Obstetrics, Hospital General San Juan de Dios, Guatemala City, Guatemala; 6 Cancer Research Technology Program, Leidos Biomedical Research, Inc., National Cancer Institute, 8560 Progress Drive, Frederick, MD 21701 USA; 7 Hospital Central Universitario “Dr. Antonio M Pineda”, Barquisimeto, Lara State Venezuela; 8 Department of Pathology, Hospital General San Juan de Dios, Guatemala City, Guatemala; 9 Medical Laboratory Services, Willemstad, Curaçao; 10 Fundashon Prevenshon, Willemstad, Curaçao

**Correction to: BMC Cancer (2018) 18:562**

**https://doi.org/10.1186/s12885-018-4438-y**

Following publication of the original article [1], an error was reported in the tagging of Joël Fokom Domgue in the author group. The tagging in this correction article has been fixed.

The current article [1] tagging is as follows:

Given Name: Joël Fokom

Family Name: Domgue

The correct tagging should be:

Given Name: Joël

Family Name: Fokom Domgue
