# Supplementary material for: Low-cost HPV testing and the prevalence of cervical infection in asymptomatic populations in Guatemala
Source: BMC Cancer. 2018 May 15;18:562. doi: 10.1186/s12885-018-4438-y (PMC5952444; doi:10.1186/s12885-018-4438-y)

**Figure S1.**

Positive: HeLa

Input DNA Amount (ng)

1. 100
2. 10
3. 1.0
4. 0.1
5. 0.01
6. 0.001
7. 0.0001
8. 0.00001

1 2 3 4 5 6 7 8

1 2 3 4 5 6 7 8

Negative: HEK293

Unknown Cell Lines

1 2 3 4 5 6 7 8 9 10 11 12

1. BL 41
2. BJAB
3. Jurkat
4. RL-95-2
5. KLE
6. MCF7
7. LNCaP
8. 22RV1
9. VCaP
10. PZHPV7
11. CAHPV10
12. RWPE1

HPV

β-globin


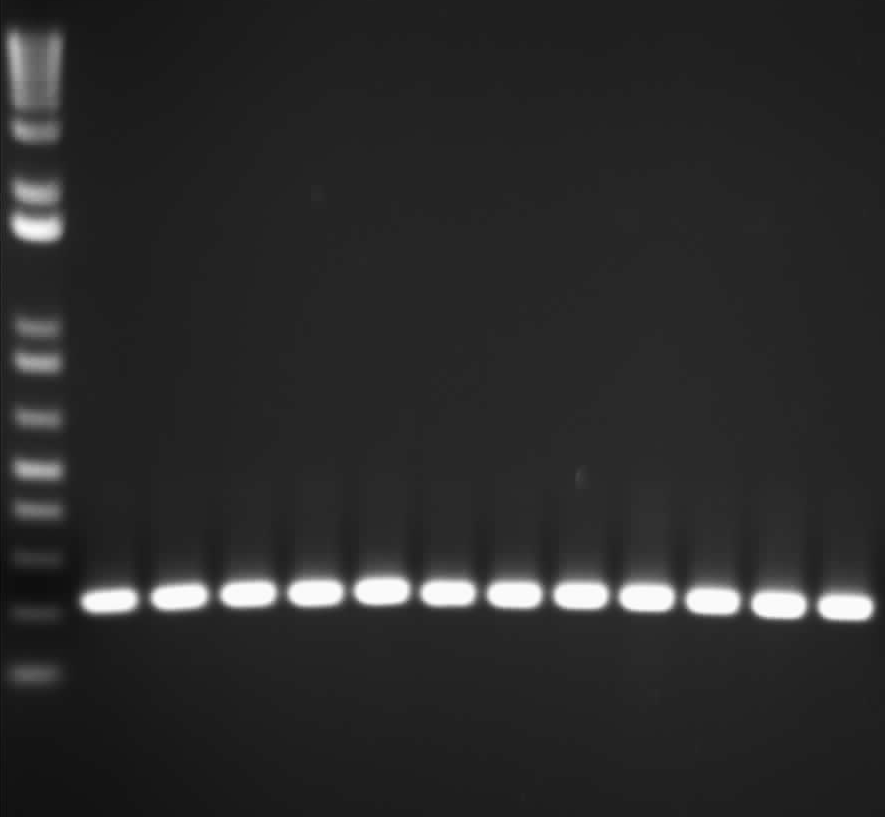

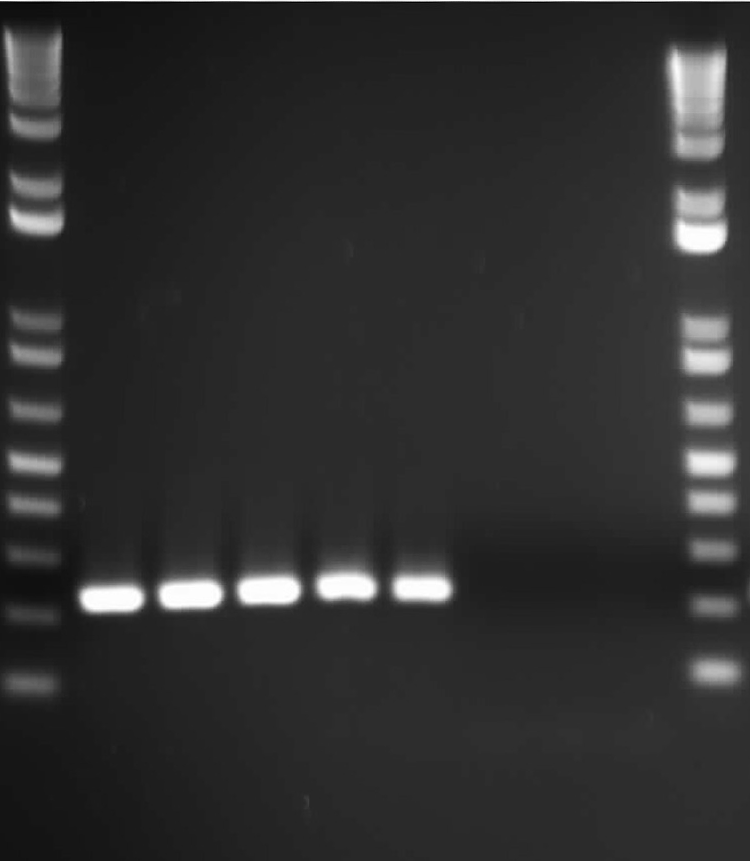

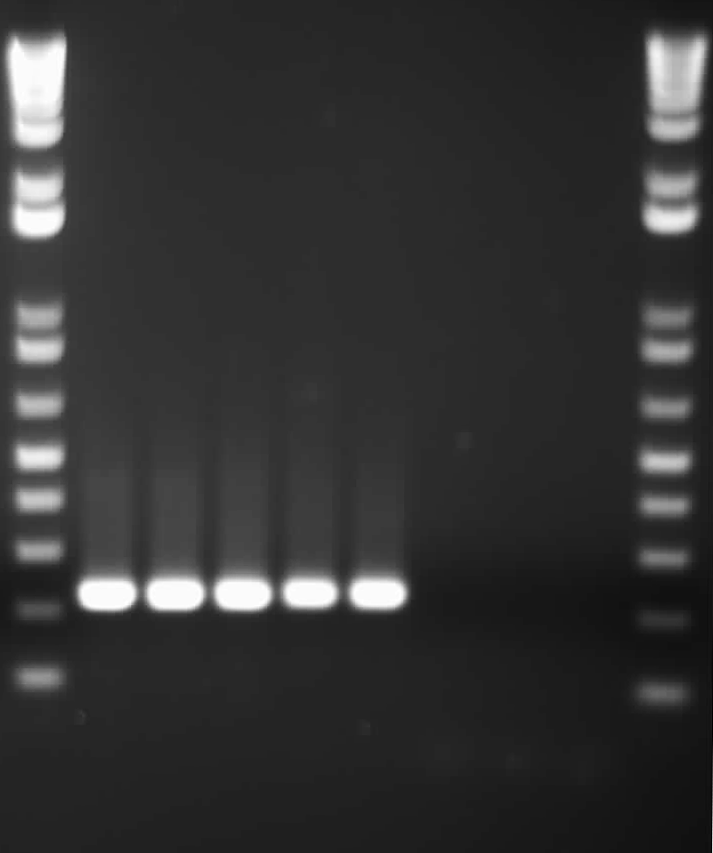


50ng / sample


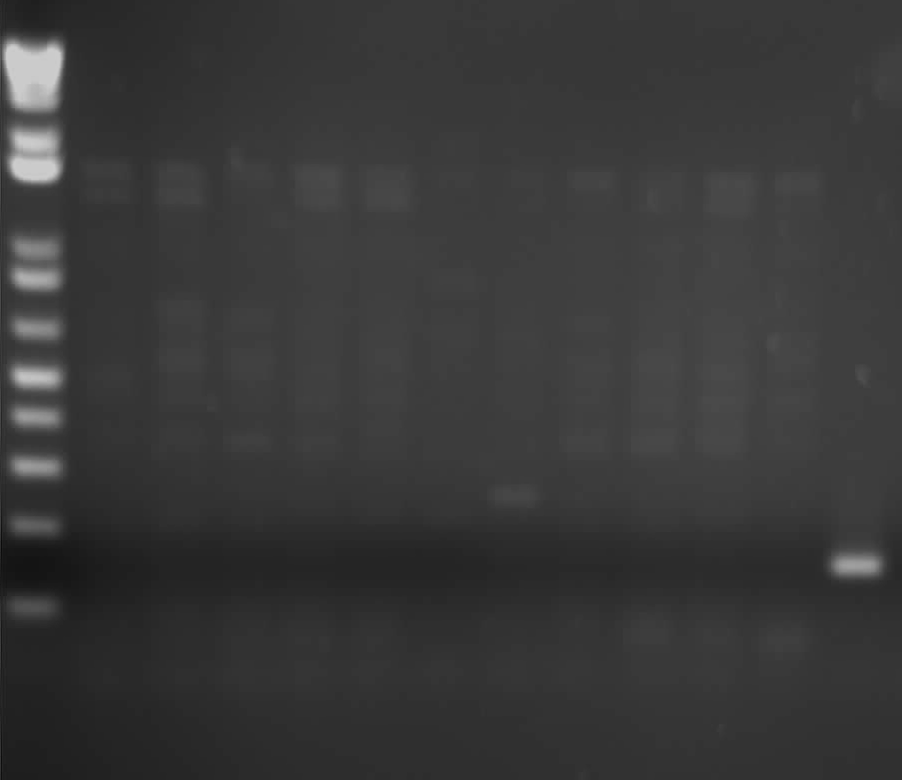

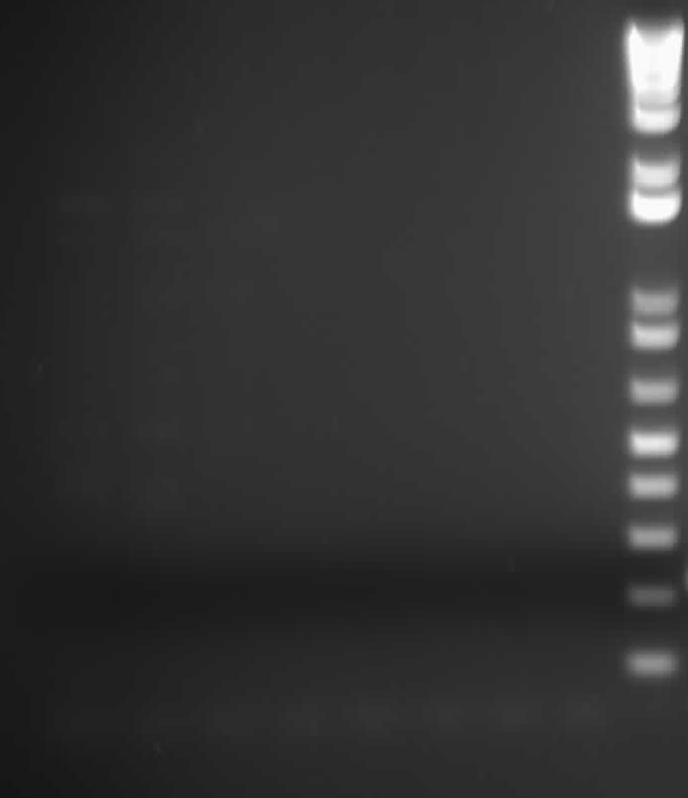

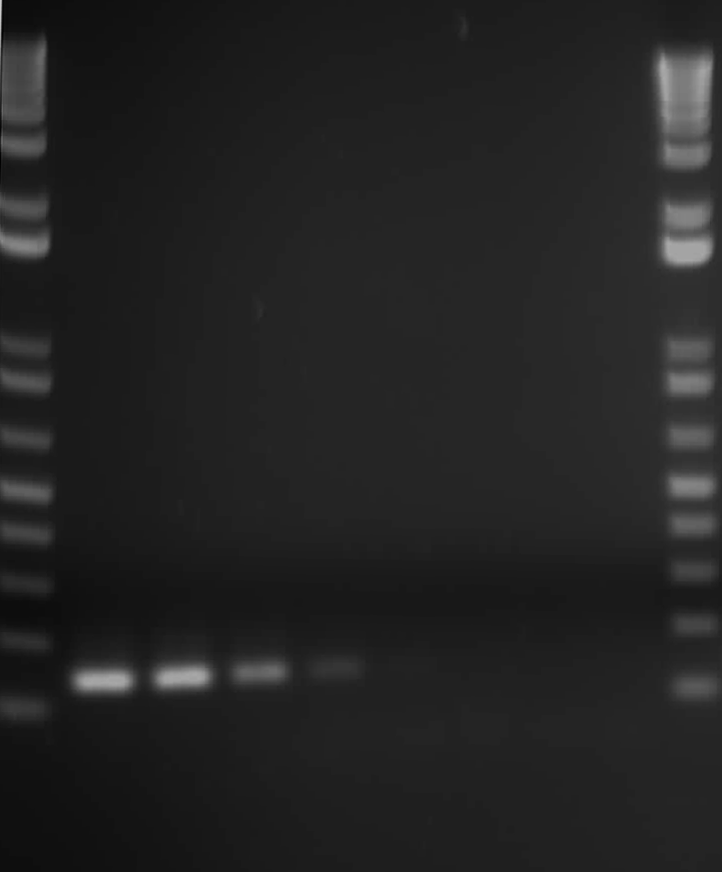


**Figure S2.**

A.

B.


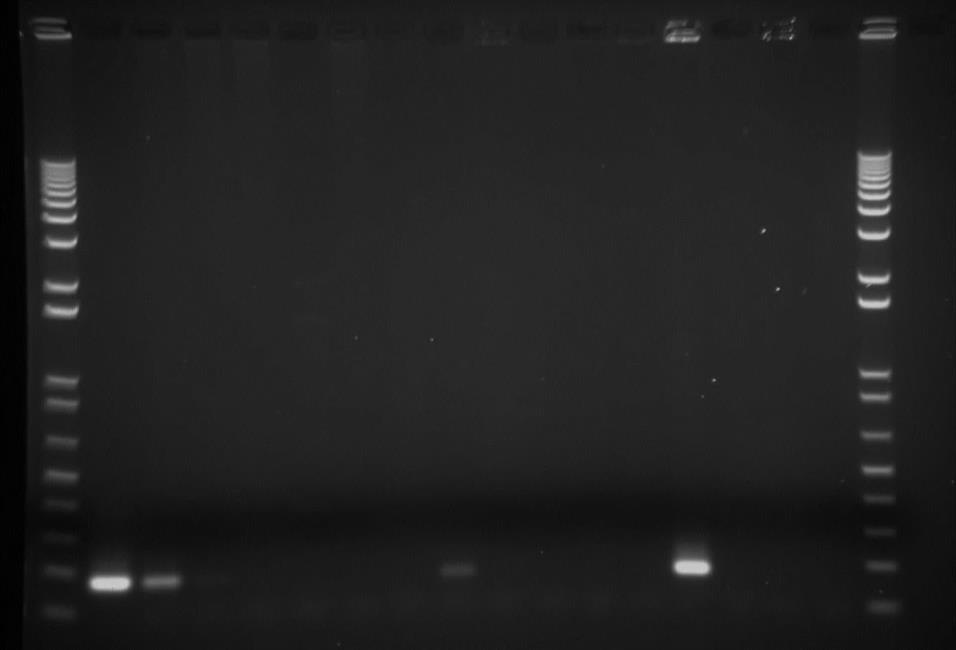


HPV

M 1 2 3 4 5 6 7 8 9 10 11 12 13 14 15 16 M


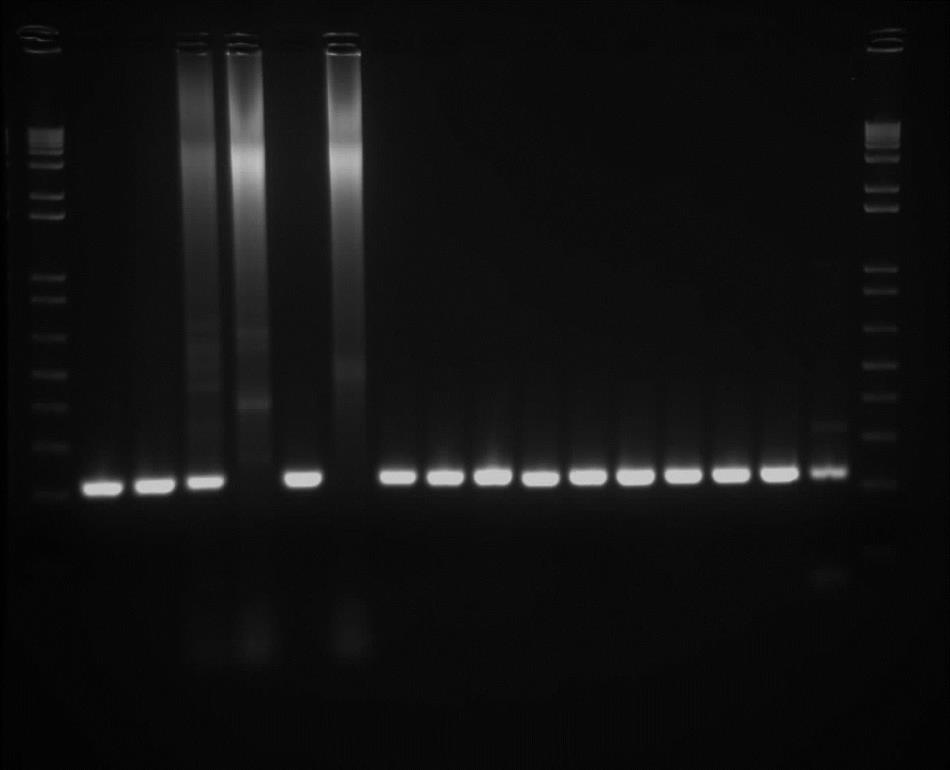


β-globin

HPV

M 1 2 3 4 5 6 7 8 9 10 11 12 13 14 15 16 17 18 M 19 20 21


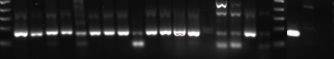

Supplement: Supplementary file 1 — Figure S1. Amplification of HPV positive and negative cell line genomic DNA by Touchdown PCR using BS GP5+/6+ primers. A dilution series of an HPV positive cell line (HeLa), HPV negative (HEK293) and HPV-status unknown cell lines were amplified by touchdown PCR with both HPV primer sets and a globin primer pair, and run on 1.5% agarose gels. Figure S2. Touchdown PCR from swab cell lysate, (A): Lane 1.- Lane 4 HPV positive control with input DNA 1 ng, 0.1 ng, 0.01 ng and 0.001 ng; Lane 5. HPV negative control (cell line); Lane 6. no template control. Lane 7 to Lane 16 indicate unknown swab samples. M indicate the DNA ladder marker. Touchdown PCR from HPV positive swabs by Hybribio Assay (B): Lane 1. – Lane 18 are swab cell lysate; Lane 19. HPV positive control (cell line); Lane 20. HPV negative control (cell line); Lane 21. no template control. The samples were amplified with the BSGP5+/6+ primers. (DOCX 2548 kb) [file 12885_2018_4438_MOESM1_ESM.docx]
